# Meningioma patients diagnosed 2007–2009 and the association with use of mobile and cordless phones: a case–control study

**DOI:** 10.1186/1476-069X-12-60

**Published:** 2013-07-19

**Authors:** Michael Carlberg, Fredrik Söderqvist, Kjell Hansson Mild, Lennart Hardell

**Affiliations:** 1 Department of Oncology, University Hospital, SE-701 85, Örebro, Sweden; 2 Department of Public Health and Community Medicine, County Council of Västmanland, SE-721 89, Västerås, Sweden; 3 Centre for Clinical Research, Uppsala University, Central Hospital Västerås, SE-721 89, Västerås, Sweden; 4 Department of Radiation Physics, Umeå University, SE-90187, Umeå, Sweden

**Keywords:** Case–control study, 25 years latency, Benign brain tumour, Meningioma, Wireless phones

## Abstract

**Background:**

To study the association between use of wireless phones and meningioma.

**Methods:**

We performed a case–control study on brain tumour cases of both genders aged 18–75 years and diagnosed during 2007–2009. One population-based control matched on gender and age was used to each case. Here we report on meningioma cases including all available controls. Exposures were assessed by a questionnaire. Unconditional logistic regression analysis was performed.

**Results:**

In total 709 meningioma cases and 1,368 control subjects answered the questionnaire. Mobile phone use in total produced odds ratio (OR) = 1.0, 95% confidence interval (CI) = 0.7-1.4 and cordless phone use gave OR = 1.1, 95% CI = 0.8-1.5. The risk increased statistically significant per 100 h of cumulative use and highest OR was found in the fourth quartile (>2,376 hours) of cumulative use for all studied phone types. There was no statistically significant increased risk for ipsilateral mobile or cordless phone use, for meningioma in the temporal lobe or per year of latency. Tumour volume was not related to latency or cumulative use in hours of wireless phones.

**Conclusions:**

No conclusive evidence of an association between use of mobile and cordless phones and meningioma was found. An indication of increased risk was seen in the group with highest cumulative use but was not supported by statistically significant increasing risk with latency. Results for even longer latency periods of wireless phone use than in this study are desirable.

## Background

Meningioma is the most common benign brain tumour and accounts for about 30% of intracranial tumours [1]. It develops from the pia and arachnoid membrane that cover the central nervous system. Meningioma is an encapsulated, well-demarcated and rarely malignant tumour. It is slowly growing and gives neurological symptoms by compression of adjacent structures. Headaches and seizures are common symptoms. This tumour type is most common among middle-aged and elderly persons. There are more women than men that develop meningioma and the incidence is about two fold higher in women than men [2,3].

Ionizing radiation is a well-established risk factor with time interval to tumour development of decades [4,5]. Sex hormones have been suggested to be of importance due to the female predominance but the role is not clear. A cohort study in Finland showed an increased risk of meningioma among postmenopausal women with ever use of estradiol-only medicine [6]. However, it has been suggested that sex hormone differences can not fully explain the higher incidence in women [7]. What the study actually shows is that the hormone receptor status does not differ between male and female meningioma. Obviously, since women have higher levels of circulating estrogens this will cause a larger growth rate and consequently a higher incidence of meningioma. In our previous study on meningioma and use of wireless phones [8] intake of oral contraceptives was no risk factor, (odds ratio (OR) = 1.0, 95% confidence interval (CI) = 0.8-1.3), whereas somewhat increased risk was found for estrogen intake (OR = 1.2, 95% CI = 0.97-1.5), to be published. We further analysed hormone treatment that started ≤ 50 years of age or > 50 years of age (approximate age of menopause) without statistically significant decreased or increased risks. The analyses were based on 916 meningioma cases and 2162 controls, cf Hardell et al. [8].

During the recent decade there has been an increase in access and ownership of wireless phones in most countries. When used they emit radiofrequency electromagnetic fields (RF-EMF). The brain is the main target organ during use of the handheld phone [9]. Thus, fear of an increased risk for brain tumours has dominated the debate during the last one or two decades. The GSM (Global System for Mobile Communication) phones and to a lesser extent the cordless phones emit also extremely low frequency magnetic field from the battery when used [10,11].

In May 2011 the International Agency for Research on Cancer (IARC) at WHO evaluated the carcinogenic effect to humans from RF-EMF. It included radiation from mobile phones, and from other devices that emit similar non-ionising electromagnetic fields in the frequency range 30 kHz – 300 GHz. It was concluded that RF-EMF is a Group 2B, i.e. a ‘possible’, human carcinogen [12,13]. The IARC decision on mobile phones was based mainly on results for glioma and acoustic neuroma in case–control studies from the Hardell group from Sweden [8,14,15] and the IARC Interphone study [16].

The IARC Working Group found for meningioma that the available evidence was insufficient to reach a conclusion on an association with mobile phone use [12]. The only studies that gave results for 10 years latency or more were those from the Hardell group [8,17] and the Interphone study group [16].

The results for meningioma as well as for other types of brain tumours are so far based on limited numbers of long-term users since the technology is fairly new. In Sweden the major increase in use (minutes of outgoing calls) and exposure to radiation fields from these phones (not merely access or ownership) in the general population is most evident after 2003 [18].

In order to get results for longer time period for use of wireless phones we decided to perform a new case–control study. Here results for benign brain tumours are presented. Updated results and discussions of this research area can be found elsewhere [19,20].

## Methods

### Wireless technology

The wireless technology has been used in Sweden since the early 1980’s. First analogue phones (NMT; Nordic Mobile Telephone System) were used, but this system was finally closed down in 2007. The market has since early 1990’s increasingly been dominated by the digital GSM phones (2G; second generation of mobile phones). In 2003 the third generation of mobile phones, 3G or UMTS (Universal Mobile Telecommunication System), was introduced in Sweden. Currently the fourth generation, 4G (Terrestrial 3G), is established. Nowadays mobile phones are used more than landline phones in Sweden [21]. Worldwide, an estimate of 5.9 billion mobile phone subscriptions were reported at the end of 2011 by the International Telecommunication Union [22].

Desktop cordless phones (DECT) have been used in Sweden since 1988, first using analogue 800–900 MHz RF fields, but since early 1990’s using a digital 1 900 MHz system. They are very common and are overtaking telephones connected to landlines. Also these devices emit RF-EMF radiation when used and should be equally much considered as mobile phones when human health risks are evaluated.

### Inclusion criteria

Our new study included both men and women aged 18–75 years at the time of brain tumour diagnosis (ICD-7 code 193.0) during 2007–2009. The diagnosis was verified by histopathology for all cases. All were alive when included in the study. They were reported to us from cancer registries and the whole of Sweden was included. For administrative reasons the Gothenburg region could only be included for the years 2008 and 2009. Sweden contains six administrative medical regions with cancer registries, which each year are linked together to the national Swedish cancer register. The reporting to us of new diagnoses of brain tumour cases varied between these six regions from once a month to once a year from one region (Umeå).

Before inclusion in the study we checked that the criteria for participation were fulfilled. After that the responsible physician was contacted for permission to include the case in the study. In Table 1 the numbers of reported cases with a benign brain tumour are displayed, in total 1,039 subjects. Of these 920 (89%) were included in the study according to the inclusion criteria.

**Table 1:** **Descriptive data on the study sample of cases with benign brain tumour diagnosed during 2007**–**2009**

	**Benign**
Reported from cancer registries	1,039
Deceased	31
Wrong diagnosis	28
Diagnosed other year	1
No address available	5
Language problems	5
Not capable to participate	20
No permission from physician	29
Total included	920
Refused to participate	106
Answered the questionnaire	814

The Swedish Population Registry was used for identification of controls. One control matched on gender and age in 5-year groups was used to each case, both with a malignant or a benign brain tumour. All controls were recruited from the same source population as the cases as soon as the treating physician had permitted inclusion of the respective case. The whole country was used for retrieving controls (Gothenburg region excluded 2007). They were assigned the same year as the diagnosis of the respective case as cut-off in assessment of exposure. The study was approved by the ethical committee: Regional Ethics Committee, Uppsala University; Uppsala, Sweden. DNR 2005:367 and the research was carried out in compliance with the Helsinki Declaration.

### Exposure assessment

Use of wireless phones, both mobile and cordless phones, was assessed by a self-administered questionnaire supplemented over the phone. There was no difference regarding supplementary interviews according to being a case (74% supplemented) or a control (70% supplemented). Adjusting for whether or not a supplementary interview was performed did not change the results of the logistic regression analysis. The questionnaire also contained a number of other questions on e.g., occupations, exposure to different agents, smoking habits, medical history including hereditary risk factors, and exposure to ionizing radiation. Also these questions were supplemented over the phone by the interviewer. A structured protocol was used for all questions. Thus, all assessed exposures were included in the questionnaire and if necessary supplemented over the phone at the same time. Results for other exposures will be published separately.

The ear that had mostly been used during calls was assessed by separate questions for mobile and cordless phones; > 50% of the time for one side, or equally much for both sides. After informed consent from the patients medical records including computer tomography (CT) and/or magnetic resonance imaging (MRI) were used for definition of tumour localisation. The matched control was assigned the same side as the tumour of the respective case. The whole procedure was done without knowledge of exposure status. Use of the wireless phone was defined as ipsilateral (≥ 50% of the time), or contralateral (< 50% of the time) in relation to tumour side.

Medical records and reports to the cancer registries were used to categorize histopathology of the tumours. In Table 2 the various diagnoses of benign brain tumours (n = 814) among participating cases are shown. Most were diagnosed with meningioma (n = 709; 87%). As expected there was a female preponderance among the cases.

**Table 2:** Histopathology of all benign brain tumours

**Histopathology**	**Men**		**Women**		**Total**	
	n	%	n	%	n	%
Meningioma	200	78.4	509	91.1	709	87.1
Pituitary adenoma	1	0.4	0	0.0	1	0.1
Acoustic neuroma	36	14.1	37	6.6	73	9.0
Hemangioblastoma	11	4.3	6	1.1	17	2.1
Other benign	7	2.7	7	1.3	14	1.7
All benign	255		559		814	

All questionnaires received a unique Id-number that did not disclose if it was a case or a control. Thus, case or control status was not disclosed to the interviewer or during the further data processing. All information was coded and entered into a database. A random sample of questionnaires was coded twice by two independent persons with similar results. Being a case or a control was not disclosed until the statistical analyses.

### Statistical methods

All analyses were done using StataSE 12.1 (Stata/SE 12.1 for Windows; StataCorp., College Station TX). Odds ratios and 95% confidence intervals were calculated using unconditional logistic regression analysis including the whole control sample (i.e. matched to both malignant and benign cases) to increase the power in the study. This was possible since adjustment/stratification was made for the matching variables (gender, age within 5 years, and year of diagnosis).

The unexposed category consisted of people who reported no use of mobile or cordless phones, or a latency period ≤ 1 year (amount of time between first use of the phone and year of diagnosis). As noted earlier, the same year as for each case’s diagnosis was used for the corresponding control as the cut-off for exposure accumulation. Furthermore, because of the low number of unexposed cases, a further criterion was used, i.e. regardless of latency being ≤1 year, cumulative use ≤ 39 hours (3^rd^ percentile) of wireless phones in total among the controls was also used as cut-off for the referent group of “no exposure” among cases and controls. The 3^rd^ percentile was chosen to approximately correspond to one working week.

A latency period ≤ 1 year was used, as in our previous studies, to make it possible to analyse a late effect (promotion) in brain tumour genesis [8,15]. Note that latency was calculated separately for the respective phone type or combination of phones that were analysed. Latency was analysed using six time periods, >1-5 years, >5-10 years, >10-15 years, >15-20 years, >20-25 years and >25 years. Cumulative use of the phone types was analysed in quartiles based on use of wireless phones in total among the controls (first quartile >39-405 h, second quartile 406–1,091 h, third quartile 1,092-2,376 h, fourth quartile >2,376 h). Latency and cumulative use were also analysed as continuous variables (per year of latency, per 100 h cumulative use) to further explore the dose–response relations.

Adjustment was made for the matching variables gender, age (as a continuous variable), and year of diagnosis. In addition, adjustment was made for socio-economic index (SEI) divided into four categories (blue-collar worker, white-collar worker, self-employed, no work). We had no information if ‘no work’ indicated unemployment, retirement, living on returns etc. Note that laterality of the tumour was not available for all cases, e.g., for midline tumours, or for tumours in both hemispheres (n = 123). These were dropped from the laterality analysis together with controls matched to cases without laterality data in the whole material (n = 306). Laterality analysis was not made for the whole group of wireless phone users since the side differed for mobile phone and cordless phone for some of the included persons using both phone types (9.8% of the cases, 8.9% of the controls).

Tumour volume was estimated using the ellipsoid formula ($\frac{4}{3}\pi \left(\frac{D_{1}}{2}\times \frac{D_{2}}{2}\times \frac{D_{3}}{2}\right)$; D_1_, D_2_, D_3_ = diameters in the three axis). Change of tumour volume per year of latency and per 100 hours of cumulative use was analysed using linear regression analysis, adjusted for age and gender. The volumes were log-transformed to normalize the distribution. The percentage changes were calculated from the β coefficients in the model, using the expression (*e*^*β* − *coefficient* − 1^) × 100.

In this article results are given for meningioma, whereas the findings for acoustic neuroma will be published separately. The number of other benign brain tumours was too low (n = 32) to make statistical analyses meaningful.

## Results

Of the 920 cases with a benign brain tumour 814 (88%) answered the questionnaire, 255 were men and 559 women. For the total sample of 1,601 cases (both malignant and benign brain tumours), an equal number of matched controls received a questionnaire. Note that two cases had two tumours; astrocytoma grade IV and meningioma and ependymoma and acoustic neuroma, respectively. Of these controls, 1,368 (85%) answered the questionnaire, 564 men and 804 women. The mean age was 56 years for cases with benign brain tumour (median 58, range 21–75) and 55 years for all controls (median 58, range 19–75). For meningioma cases the mean age was 57 years (median 59, range 23–75).

In Table 3 the results are shown for meningioma and use of wireless phones. Analogue phones yielded OR = 0.9, 95% CI = 0.6-1.5 and OR = 1.3, 95% CI = 0.6-2.8 in the longest latency group > 25 years.

**Table 3:** **Odds ratio** (**OR**) **and 95% confidence interval** (**CI**) **for meningioma**

**Latency**	**Analogue**	**Digital(2G)**	**Digital(UMTS,3G)**	**Mobile phone,** **total**	**Cordless phone**	**Digital type**^**a**^	**Wireless phone**
	OR, CI	OR, CI	OR, CI	OR, CI	OR, CI	OR, CI	OR, CI
	(Ca/Co)	(Ca/Co)	(Ca/Co)	(Ca/Co)	(Ca/Co)	(Ca/Co)	(Ca/Co)
**Meningioma** (**n** = **709**)							
Total, > 1 y	0.9	1.0	0.7	1.0	1.1	1.0	1.0
	0.6-1.5	0.7-1.4	0.4-1.2	0.7-1.4	0.8-1.5	0.7-1.5	0.7-1.5
	(108/260)	(593/1,208)	(47/140)	(594/1,217)	(522/1,015)	(641/1,261)	(641/1,261)
>1-5 y	-	1.1	0.6	1.1	1.0	1.2	1.2
		0.7-1.7	0.3-1.2	0.7-1.7	0.7-1.5	0.7-1.9	0.7-2.0
	(0/0)	(70/109)	(40/126)	(69/108)	(109/209)	(43/64)	(42/61)
>5-10 y	0.5	0.9	1.1	1.0	1.0	1.0	1.0
	0.1-2.1	0.7-1.4	0.4-3.5	0.7-1.4	0.7-1.5	0.7-1.4	0.7-1.5
	(3/10)	(236/477)	(7/14)	(217/423)	(217/436)	(222/420)	(206/378)
>10-15 y	0.8	1.0	-	1.0	1.1	1.0	1.0
	0.4-1.6	0.7-1.5		0.7-1.4	0.8-1.7	0.7-1.5	0.7-1.5
	(21/51)	(212/453)	(0/0)	(185/399)	(128/248)	(248/523)	(226/466)
>15-20 y	1.1	1.0	-	1.0	1.2	1.1	1.1
	0.6-1.9	0.6-1.5		0.6-1.5	0.7-1.8	0.7-1.6	0.7-1.6
	(39/86)	(75/169)	(0/0)	(78/174)	(61/109)	(121/241)	(115/231)
>20-25 y	0.9	-	-	0.8	1.3	1.2	0.9
	0.5-1.5			0.5-1.4	0.5-3.4	0.5-3.3	0.5-1.5
	(29/80)	(0/0)	(0/0)	(29/80)	(7/13)	(7/13)	(36/92)
>25 y	1.3	-	-	1.2	-	-	1.2
	0.6-2.8			0.6-2.3			0.6-2.4
	(16/33)	(0/0)	(0/0)	(16/33)	(0/0)	(0/0)	(16/33)

Number of exposed cases (Ca) and controls (Co) are given.

Adjustment was made for age at diagnosis, gender, SEI-code and year of diagnosis.

^a^2G, 3G and/or cordless phone.

Use of digital 2G phones yielded in total OR = 1.0, 95% CI = 0.7-1.4. Similar results were found in the different latency group, i.e. no increased risk. Also for digital 3G phones no statistically significant increased risk was found as well as for mobile phone use in total.

Cordless phone use gave OR = 1.1, 95% CI = 0.8-1.5, with somewhat higher risk in the longest latency group >20-25 years yielding OR = 1.3, 95% CI = 0.5-3.4. Wireless phone use overall gave OR = 1.0, 95% CI = 0.7-1.5 increasing somewhat with latency > 25 years to OR = 1.2, 95% CI = 0.6-2.4. Gender specific analyses did not change the results statistically significant (data not in table).

In Table 4 results are given for use of wireless phones in relation to tumour side. The results were similar for ipsilateral and contralateral use without any statistically significant increased or decreased risk for the different phone types.

**Table 4:** **Odds ratio** (**OR**) **and 95**% **confidence interval** (**CI**) **for meningioma**, **total**, **ipsilateral and contralateral exposure**

	**All**			**Ipsilateral**			**Contralateral**		
	Ca/Co	OR	95% CI	Ca/Co	OR	95% CI	Ca/Co	OR	95% CI
Analogue	108/260	0.9	0.6 – 1.5	54/118	1.4	0.8 – 2.4	42/84	1.2	0.6 – 2.2
Digital (2G)	593/1,208	1.0	0.7 – 1.4	283/530	1.1	0.7 – 1.6	214/404	1.1	0.7 – 1.6
Digital (UMTS, 3G)	47/140	0.7	0.4 – 1.2	26/69	0.8	0.4 – 1.8	17/45	0.8	0.3 – 2.1
Mobile phone, total	594/1,217	1.0	0.7 – 1.4	284/534	1.1	0.7 – 1.6	214/407	1.1	0.7 – 1.6
DECT	522/1,015	1.1	0.8 – 1.5	244/454	1.1	0.7 – 1.6	188/327	1.2	0.8 – 1.8

Numbers of exposed cases (Ca) and controls (Co) are displayed.

Adjustment was made for age at diagnosis, gender, SEI-code and year of diagnosis.

Ipsilateral: ≥ 50% use of the phone on the same side as the tumour was located.

Contralateral: < 50% use of the phone on the same side as the tumour was located.

Cumulative use of wireless phones was analysed in quartiles, Table 5. Note that for the various phone types the cumulative time was counted for use of the specific phone, but for the category “mobile phones” all types of mobile phones were included, and for “wireless phones” also use of cordless phones was included. For all studied phone types and combinations highest ORs were found in the fourth quartile with > 2,376 h cumulative use. Mobile phone use gave OR = 1.3, 95% CI = 0.8-1.9 (p trend = 0.34), cordless phone use yielded OR = 1.8, 95% CI = 1.2-2.8 (p trend = 0.0003) and wireless phone use in total gave OR = 1.4, 95% CI = 0.9-2.0 (p trend = 0.01).

**Table 5:** **Odds ratio** (**OR**) **and 95**% **confidence interval** (**CI**) **for meningioma for cumulative use of wireless phones in quartiles based on use of wireless phones among controls in total**

**Quartile**	**Analogue**	**Digital(2G)**	**Digital(UMTS,3G)**	**Mobile phone,total**	**Cordless phone**	**Digital type**	**Wireless phone**
	OR, CI	OR, CI	OR, CI	OR, CI	OR, CI	OR, CI	OR, CI
	(Ca/Co)	(Ca/Co)	(Ca/Co)	(Ca/Co)	(Ca/Co)	(Ca/Co)	(Ca/Co)
First quartile	0.9	1.0	0.7	1.0	1.0	1.1	1.1
	0.6-1.5	0.7-1.4	0.3-1.3	0.7-1.4	0.7-1.4	0.8-1.6	0.7-1.5
	(77/184)	(317/620)	(30/87)	(306/587)	(194/434)	(185/327)	(178/317)
Second quartile	0.6	1.0	0.4	1.0	0.9	0.9	0.9
	0.3-1.4	0.7-1.5	0.1-1.2	0.7-1.4	0.6-1.3	0.6-1.3	0.6-1.3
	(12/47)	(122/260)	(6/34)	(119/261)	(116/278)	(134/320)	(134/314)
Third quartile	1.3	0.9	0.6	0.9	1.2	0.9	0.9
	0.6-2.9	0.6-1.4	0.2-1.8	0.6-1.4	0.8-1.8	0.6-1.3	0.6-1.4
	(12/23)	(75/199)	(6/17)	(85/210)	(117/194)	(135/317)	(138/315)
Fourth quartile	3.0	1.5	7.3	1.3	1.8	1.4	1.4
	0.9-9.7	0.9-2.3	1.2-46	0.8-1.9	1.2-2.8	0.96-2.0	0.9-2.0
	(7/6)	(79/129)	(5/2)	(84/159)	(95/109)	(187/297)	(191/315)
p, trend	0.11	0.06	0.04	0.34	0.0003	0.002	0.01

Numbers of exposed cases (Ca) and controls (Co) are displayed.

Adjustment was made for age at diagnosis, gender, SEI-code and year of diagnosis.

First quartile >39-405 h; second quartile 406–1,091 h; third quartile 1,092–2,376 h, fourth quartile >2,376 h.

OR increased per 100 h cumulative use, statistically significant for all types of phones except for 2G with borderline significance, Table 6. In a multivariate analysis including all phone types (i.e. analogue, 2G, 3G and cordless phone) a statistically significant result was found only for cordless phone (OR = 1.010, 95% CI = 1.005-1.016; data not in table). Wireless phone use increased the risk with OR = 1.006, 95% CI = 1.003-1.009 per 100 h cumulative use. Regarding OR per year of latency no statistically significant increased risk was found. These results did not change if years of use of any mobile or cordless phone prior to the respective type was included as a covariate in each analysis of the individual phone types (data not in table).

**Table 6:** **Odds ratio** (**OR**) **and 95**% **confidence interval** (**CI**) **for meningioma per 100 hours of cumulative use and per year of latency**

	**Per 100 h cumulative use**		**Per year of latency**	
	OR	95% CI	OR	95% CI
Analogue	1.021	1.0004 – 1.042	1.003	0.982 – 1.025
Digital (2G)	1.005	0.99997 – 1.011	0.999	0.979 – 1.020
Digital (UMTS, 3G)	1.035	1.0002 – 1.071	0.929	0.799 – 1.081
Mobile phone, total	1.005	1.001 – 1.010	0.998	0.982 – 1.014
Cordless phone	1.011	1.006 – 1.017	1.008	0.989 – 1.028
Digital type	1.007	1.003 – 1.010	1.003	0.984 – 1.022
Wireless phone	1.006	1.003 – 1.009	1.000	0.984 – 1.016

Adjustment was made for age at diagnosis, gender, SEI-code and year of diagnosis.

In Table 7 results are shown for malignant brain tumours localized in the temporal lobe or overlapping temporal and adjacent lobe. There was no pattern of statistically significant increased risk for any phone type in total or in the different latency groups.

**Table 7:** **Odds ratio** (**OR**) **and 95**% **confidence interval** (**CI**) **for meningioma located in temporal** (**n** = **169**) **and overlapping lobes** (**temporofrontal** (**n** = **44**), **temporoparietal** (**n** = **11**), **temporooccipital** (**n** = **5**)); **in total n** = **229**

**Latency**	**Analogue**	**Digital(2G)**	**Digital(UMTS,3G)**	**Mobile phone,total**	**Cordless phone**	**Digital type**	**Wireless phone**
	OR, CI	OR, CI	OR, CI	OR, CI	OR, CI	OR, CI	OR, CI
	(Ca/Co)	(Ca/Co)	(Ca/Co)	(Ca/Co)	(Ca/Co)	(Ca/Co)	(Ca/Co)
Total, > 1 y	1.0	0.8	0.9	0.8	0.9	0.9	0.9
	0.5-1.9	0.5-1.4	0.4-2.1	0.5-1.4	0.5-1.5	0.5-1.5	0.5-1.5
	(35/260)	(188/1,208)	(20/140)	(188/1,217)	(170/1,015)	(205/1,261)	(205/1,261)
>1-5 y	-	0.9	0.9	0.9	0.8	1.1	1.2
		0.5-1.7	0.4-2.2	0.4-1.7	0.5-1.5	0.6-2.3	0.6-2.4
	(0/0)	(21/109)	(19/126)	(21/108)	(33/209)	(16/64)	(16/61)
>5-10 y	-	0.8	0.5	0.8	1.0	0.8	0.8
		0.5-1.3	0.1-5.2	0.4-1.3	0.6-1.6	0.5-1.4	0.5-1.3
	(0/0)	(71/477)	(1/14)	(64/423)	(75/436)	(64/420)	(59/378)
>10-15 y	1.0	0.9	-	0.9	0.9	0.9	0.9
	0.4-2.6	0.5-1.6		0.5-1.5	0.5-1.6	0.6-1.6	0.5-1.5
	(7/51)	(72/453)	(0/0)	(61/399)	(40/248)	(85/523)	(72/466)
>15-20 y	1.1	0.8	-	0.9	0.9	0.9	1.0
	0.4-2.5	0.4-1.6		0.5-1.7	0.5-1.8	0.5-1.6	0.5-1.7
	(12/86)	(24/169)	(0/0)	(26/174)	(19/109)	(37/241)	(39/231)
>20-25 y	1.0	-	-	0.9	1.1	1.2	1.0
	0.4-2.3			0.4-2.0	0.3-4.4	0.3-4.8	0.5-2.0
	(11/80)	(0/0)	(0/0)	(11/80)	(3/13)	(3/13)	(14/92)
>25 y	1.1	-	-	1.0	-	-	1.0
	0.4-3.6			0.4-3.0			0.4-3.0
	(5/33)	(0/0)	(0/0)	(5/33)	(0/0)	(0/0)	(5/33)

Numbers of exposed cases (Ca) and controls (Co) are given.

Adjustment was made for age at diagnosis, gender, SEI-code and year of diagnosis.

The average tumour volume in men was 32.6 cm^3^ and 28.7 cm^3^ in women (p = 0.02). In cases with wireless phone use the average volume was 29.3 cm^3^*versus* 34.9 cm^3^ in the unexposed group (p = 0.11). Tumour volume did not change statistically significant per year of latency or per 100 hours of cumulative use, see Table 8. We calculated also tumour area and found no statistically significant association with cumulative use or latency for wireless phone use (data not in table).

**Table 8:** Percentage change in tumour volume per year of latency and per 100 hours of cumulative use

**Type of phone**	**n**	**Change in volume per year of latency (%)**	**95%CI**	**p**	**Change in volume per 100 h of cumulative use (%)**	**95%CI**	**p**
Analogue	98	1.6	−4.7 to 8.3	0.62	0.1	−2.0 to 2.2	0.96
Digital, 2G	530	−0.9	−4.0 to 2.2	0.56	0.1	−0.6 to 0.8	0.83
Digital, UMTS, 3G	41	9.6	−21.1 to 52.4	0.57	1.3	−2.0 to 4.7	0.42
Mobile phone, total	531	−0.5	−2.8 to 1.9	0.68	0.1	−0.5 to 0.6	0.84
DECT	465	−0.8	−3.6 to 2.0	0.57	−0.3	−0.7 to 0.1	0.13
Wireless phone	570	−0.2	−2.5 to 2.1	0.86	−0.2	−0.5 to 0.1	0.19

Adjustment was made for age at diagnosis and gender.

## Discussion

The main result of this study was no overall association between use of wireless phones and meningioma. However, somewhat higher OR was found in the longest latency group, > 25 years, for use of analogue phones. A similar result was found for use of cordless phones in the latency group > 20–25 years, the longest time for that phone type. These results were not statistically significant and no statistically significant increased OR was calculated per year of latency.

The highest absorption of RF-EMF emissions from a handheld phone is on the same side of the brain (ipsilateral) as the phone is used, with highest dose in the temporal lobe [9]. In the present study there was no effect of laterality, although somewhat higher OR was calculated for ipsilateral use of an analogue phone than contralateral. No pattern of association was found for meningioma in the temporal and overlapping lobes.

Cumulative use of wireless phones was in our present study divided into quartiles depending on cumulative use of wireless phones in total among controls. For all phone types the highest risk was found in the fourth quartile > 2,376 hours of cumulative use. This corresponds to about 40 min wireless phone use per day for 10 years. There was a statistically significant trend (p < 0.05) for increasing cumulative use of 3G mobile phones, cordless phones, phones of the digital type (2G, 3G and/or cordless phone), and wireless phones in total. Especially high OR was calculated for digital 3G phone use, OR = 7.3, 95% CI = 1.2-46, in the fourth quartile, but based on only 5 exposed cases and 2 exposed controls. These results are reflected in Table 6 with a statistically significant increasing risk per 100 h cumulative use for all phone types except for 2G with borderline statistical significance.

Tumour volume was not statistically significant associated with use of wireless phones. However, meningioma grows to a size that depends on the location until symptoms. If pressure of the tumor induces symptoms (e.g. seizures, headache) it might be detected sooner and at a smaller volume than in areas where symptoms might remain unnoticed or not being related to a tumor for a long time. If mobile phone use increases tumor growth rate this might be associated with a larger volume but with earlier diagnosis. To elucidate that possibility to some extent we analysed tumour volume for meningioma located in temporal and adjacent lobes, frontal lobe, and other localisations. No clear trends were found for either of these locations with respect to change in volume per year of latency and per 100 h of cumulative use (data not in table).

There are some strengths of the study. Cases from the whole Sweden with a benign brain tumour diagnosed during 2007–2009 were included. The prevalence of use of mobile phones was highest in the age group 30–54 years for men and 35–54 years for women for the cases diagnosed during 1997–2003 in our previous study [19]. Thus, we included the age group 18–75 years in this study to allow for a reasonable latency time [23]. This is in contrast to the Interphone study that only included cases aged 30–59 years old.

We included only cases with a histopathological diagnosis of a brain tumour. Hence, we asked the six regional cancer registries not to report cases with only a clinical diagnosis. The reason was that we wanted to get a valid diagnosis of the brain tumour for separate analysis depending on the tumour type. If necessary the histopathological reports were supplemented by records from pathology departments around the country after informed consent from the case. Thus, we were able to make classification of all brain tumours based on WHO codes, see Table 2. It is not probable that exclusion of cases with only clinical diagnosis would have biased the results, since criteria for diagnosis are not expected to be related to habits of wireless phone use.

An advantage of this study was the fairly high response rate among both cases and controls. The response rate was 88% (n = 814) among the finally included cases with benign brain tumour. Of the controls 85% (n = 1,368) answered the questionnaire. These response rates are similar to our previous studies on benign brain tumours, 88% (n = 1,254) among cases and 89% (n = 2,162) among controls [8]. Lower response rates were obtained in the Interphone study especially for controls; meningioma cases 78%, range by centre 56–92%, (n = 2,425), and controls 53%, range 42–74%, (n = 7,658) for controls [16]. To get as valid results as possible it is always necessary to have a high response rate. In fact, not responding controls in Interphone tended to be less frequent users of mobile phone than participating controls leading to underestimation of the risk [24-26].

In the unconditional logistic regression analysis all controls, both to cases with malignant and benign brain tumour, were used so as to maximise the statistical power. This was possible since adjustment was made for the matching variables age, gender, and year of diagnosis. In addition adjustment was made for socioeconomic index since an association between white-collar work and brain tumours has been reported [27]. Analysis using conditional logistic regression yielded overall for wireless phones OR = 1.1, 95% CI = 0.7-1.6 versus OR = 1.0, 95% CI = 0.7-1.5 using unconditional logistic regression (see Table 3). Similar differences were seen for the different phone types i.e. similar estimates using both methods, although with slightly wider confidence intervals in the conditional logistic regression.

One limitation of the study was that it was not possible to obtain an “unexposed” group with enough numbers for meaningful statistical calculations, since practically everybody is using a wireless phone of some kind today. We therefore in addition to latency ≤ 1 year used the 3^rd^ percentile (39 h) of cumulative time as cut-off. Another option to obtain more "unexposed" individuals would have been to change the cut-off for latency. However, doing that would limit the possibility to study a late effect (promotion) in brain tumour genesis. Furthermore it is difficult to find users that have been using only one single technology, i.e. NMT, GSM, UMTS etc. Most users have used several technologies and for example regarding 3G phones only one case stated use of only that type of mobile phone and no case or control has used only analogue phones. Thus, few users hampered statistical analyses of single types of wireless phones.

In our previous studies we have only included living cases so as to get as good assessment of exposure as possible [8,14,28]. Excluding deceased cases might theoretically bias the results, notably if there is no association between use of wireless phones and brain tumour in that patient group or even a protective effect. However, in the present study only 31 cases were deceased so it is unlikely that the results were biased in that respect.

Ionizing radiation is an established risk factor for brain tumours, generally more strongly associated with meningioma than with glioma. Among atomic bomb survivors a greatly increased risk for meningioma has been found, as well as among children with radiation therapy for scalp ringworm [4]. In a review of estimated exposure doses to the brain in eight cohort studies no effect modification on the risk by sex, age at exposure, time since exposure or attained age was observed [5]. In a study on radiation associated tumours following therapeutic cranial radiation there was a positive association between dose of cranial irradiation and development of meningioma with mean latency 21.8 years [29]. Average time interval may be dependent on dose, and intervals to tumour appearance of 35, 26 and 19–24 years have been reported for low-, moderate-, and high-dose radiation, respectively [30]. Thus, regarding RF-EMF emissions and an association with meningioma long latency times of decades would be expected. In previous studies results for longest latency times of 10+ years have been displayed.

In our previous study on meningioma [8] diagnostic X-ray of the head and neck was associated with an overall increased risk; OR = 1.9, 95% CI = 1.5-2.4 (to be published). The risk increased to OR = 4.4, 95% CI =2.4-8.2 for ≥ 3 times of X-rays using > 1 year latency. However, there was no interaction with mobile phone use (p = 0.52), cordless phone use (p = 0.27), or wireless phone use (p = 0.51). Also in the present study X-ray investigations of the head and neck were assessed. These data are to be further analysed, but based on our previous results it is unlikely that there is an interaction with wireless phone use.

In Interphone statistically significant decreased meningioma risk with OR = 0.79, 95% CI = 0.68-0.91 was reported overall [16]. No effect modification was found for time since start of use. With cumulative call time > 1,640 hours the risk increased somewhat to OR = 1.15, 95% CI = 0.81-1.62. We have discussed the many shortcomings in Interphone elsewhere [19,26].

In the Hardell group study for the time period 1997–2003 somewhat increased risk was found for meningioma in the > 10 year latency group for use of analogue and digital mobile phones and for use of cordless phones. Also ipsilateral use gave somewhat increased risk [8]. Wireless phone in total gave OR = 1.0, 95% CI = 0.9-1.2 increasing to OR = 1.4, 95% CI = 0.97-2.0 in the > 10 years latency group with similar results for both mobile phone and cordless phone [20]. In the present study wireless phone use in total yielded OR = 1.0, 95% CI = 0.7-1.5 with an identical result in the > 10 years latency group (data not in table).

Meta-analysis of use of mobile phones based on the results in Interphone [16] and the Hardell group [8] gave no statistically significant decreased or increased risk [19]. Somewhat increased risk was found for meningioma in the temporal lobe using latency time of ≥ 10 years (> 10 years in the Hardell group) with OR = 1.25, 95% CI = 0.31-4.98. Cumulative use ≥ 1640 hours yielded OR = 1.35, 95% CI = 0.81-2.23 for ipsilateral use of mobile phone. However, for the most exposed area, temporal lobe, OR = 0.82, 95% CI = 0.31-2.17 was calculated for ≥ 1,640 hours of cumulative use [19]. Thus, no consistent pattern of an association was found.

## Conclusions

No conclusive evidence of an association between use of mobile and cordless phones and meningioma was found in this study. The results are in agreement with previous findings of no consistent evidence of an association between use of mobile and cordless phones and meningioma. The present results strengthen our previous findings of an increased risk for glioma and acoustic neuroma, since a systematic bias in those studies would have been expected also in this study of meningioma using the same methodology. An indication of increased risk for meningioma was seen in the group with highest cumulative use but was not supported by statistically significant increasing risk with latency. However, considering the long latency periods that have been reported for the increased meningioma risk associated with exposure to ionizing radiation it is still too early to make a definitive risk assessment. Results for even longer latency periods of wireless phone use than in this study are desirable.

## Consent

Written informed consent was obtained from the subjects for the publication of this report.

## Abbreviations

2G: Second generation of mobile phones (GSM); 3G: Third generation of mobile phones (UMTS); 4G: Fourth generation of mobile phones; CI: Confidence interval; CT: Computer tomography; DECT: Desktop cordless phones; GSM: Global System for Mobile Communication; IARC: International Agency for Research on Cancer; MRI: Magnetic resonance imaging; NMT: Nordic Mobile Telephone System; OR: Odds ratio; RF-EMF: Radiofrequency electromagnetic fields; SEI: socio-economic index; UMTS: Universal Mobile Telecommunication System.

## Competing interests

The authors declare that they have no competing interests.

## Authors’ contributions

MC made the statistical calculations and LH was responsible for drafting of the manuscript. FS and KHM read and gave valuable comments on the manuscript. All authors have read and approved the final version.
